# Community engagement to address socio-ecological barriers to physical activity among African American breast cancer survivors

**DOI:** 10.21633/jgpha.6.312

**Published:** 2017

**Authors:** Selina A. Smith, Mary S. Whitehead, Joyce Q. Sheats, Brittney Chubb, Ernest Alema-Mensah, Benjamin E. Ansa

**Affiliations:** 1 Department of Family Medicine, Medical College of Georgia, Augusta, GA; 2 SISTAAH Talk Breast Cancer Support Group, Miami, FL; 3 Institute of Public and Preventive Health, Augusta, GA; 4 Department of Community Health and Preventive Medicine, Atlanta, GA

**Keywords:** Community engagement, social ecological framework, physical activity, behavior, African American, breast cancer survivor, cancer prevention guidelines

## Abstract

**Background:**

With high rates of obesity, low levels of physical activity (PA), and lack of adherence to physical activity guidelines (PAGs) among African American (AA) breast cancer survivors (BCSs), culturally appropriate interventions that address barriers to participation in PA are needed.

**Methods:**

To develop intervention content, members of an AA breast cancer support group participated in four 1-hour focus group discussions (related to the barriers to PA, strategies for overcoming them, and intervention content), which were audiotaped, transcribed, and analyzed.

**Results:**

The support group collaborated with researchers to construct the Physical Activity Intervention Developed (PAID) to Prevent Breast Cancer, a multi-component (educational sessions; support group discussions; and structured, moderately intensive walking, strength training, and yoga), facilitated, 24-week program focused on reducing multi-level barriers to PA that promote benefits (‘pay off’) of meeting PAGs.

**Conclusions:**

Community engagement fostered trust, promoted mutuality, built collaboration, and expanded capacity of AA BCSs to participate in developing an intervention addressing individual, interpersonal, organizational, and community barriers to PA.

## INTRODUCTION

Compared to Whites, AA BCSs are less physically active and more sedentary (Behavioral Risk Factor Surveillance System, 2017), less likely to adhere to PAGs (American Cancer Society/ACS, 2017), and have larger reductions in breast cancer risk from PA (Ballard-Barbash et al., 2013). To reduce these disparities, community partnership approaches are needed to develop effective interventions.

Community engagement involves working collaboratively with groups of people affiliated by similar situations to address issues affecting their well-being (Centers for Disease Control and Prevention, 1997). In this brief report, we outline the process and results of engaging AA BCSs in developing a PA intervention.

## METHODS

Participation in support groups may foster hope and offer emotional assistance, confidence, and strength, and thereby lead to improved coping, less distress, and an enhanced quality of life (Sears et al., 2003). They may be an untapped, indigenous resource for promoting PA.

Founded in 1995, SISTAAH (Survivors Involving Supporters to Take Action in Advancing Health) Talk has a mission of providing a forum for AA women to communicate about and make sense of their breast cancer experience in order to achieve improved physical and mental health outcomes. This support group has partnered with researchers to complete studies and publish findings, including the present report.

To undergird the process of engaging SISTAAH Talk members to develop a support group-based, multi-component, community intervention addressing barriers that prevent AA BCSs from participating in PA to meet PAGs, the conceptual framework included: 1) community coalition action theory (Butterfoss, et al., 2002), which posits pooling abilities, expertise, and stakeholder resources to positively affect community health; 2) social ecological perspectives, which influence PA barriers and behaviors across various levels (McElroy et al., 1988); and 3) science-based PAGs to prevent cancer, which may also prevent recurrence (ACS, 2017; World Cancer Research Foundation/American Institute for Cancer Research, 2007).

The Institutional Review Board of Augusta University approved this study, and participant consent was obtained prior to enrollment. SISTAAH Talk members participated in four 1-hour focus group discussions (FGDs) led by a BCS trained in qualitative assessments. Each FGD concentrated on barriers to PA and the cultural appropriateness, comprehension of health messages, length, and planned delivery format of the education and exercise sessions.

The FGDs were digitally recorded, transcribed verbatim, manually coded, and summarized. NVIVO 10 software (2015) was used to facilitate the coding process. Data were analyzed using qualitative content analysis. Recurring themes were identified and summarized.

## RESULTS

For participants (n=60; mean age 45.73 years; SD 7.91; range 35–75 years old), there were 4 FGDs, with findings organized into categories: 1) identification of barriers, 2) recommendation of strategies, and 3) selection of exercises. Identified barriers (Table 1) were classified based on a social ecological framework (Joseph et al., 2015): individual (post-treatment symptoms, fatigue, post-treatment body image, competing priorities, co-morbidity, PA perceptions); interpersonal (lack of family and social support, intimate partner concerns); organizational (PA preferences, monetary costs, cultural appropriateness); and community (facilities, weather, safety).

To address PA barriers, the Physical Activity Intervention Developed (PAID) to Prevent Breast Cancer included three components:

1. Didactic Instructions with strategies to enhance PA presented by PowerPoint, printed fact sheets, and SISTAAH Talk workout videos. The education sessions (Table 2) include components of the social cognitive theory (SCT) (Murrock et al., 2009): self-efficacy (the belief that one is capable of meeting PAGs); outcome expectations (e.g., physical, social, and self-evaluative), linked to greater adherence to PAGs, including desired physical changes (e.g., improved body weight); opportunities for socialization (e.g., social support); and self-worth (e.g., goal setting and self-monitoring).
2. Support Group Discussions to provide social support, monitor progress, and provide/receive feedback. Exchanges that occur during support group discussions will address barriers to PA. Each will consist of interactive presentations, demonstrations, and guest speakers, and will provide an open forum for sharing experiences, obtaining advice, accessing resources, and gaining support for PA.
3. Exercise Sessions with an experiential engaged approach. To achieve a program of structured, moderate-intensity PA aimed at meeting the PAGs, BCSs selected three exercises:
  - Walking at various levels based on capability (power, speed interval, strength interval, walking-to-jogging, and stretching)
  - Yoga to address fatigue and poor physical functioning (physical postures, conscious breathing, and meditation)
  - Strength training using lightweight dumbbells with guidance on maintaining safety

## DISCUSSION/CONCLUSIONS

This report describes a process of developing a PA intervention for AA BCSs using a community-engaged approach. Members of a breast cancer support group, mean age 45.7 years, 1) participated in four 1-hour FGDs, with findings organized as individual, interpersonal, organizational, and community barriers; 2) developed culturally tailored strategies (in 24 educational sessions); and 3) selected three exercises (walking, strength training, and yoga) to meet PAGs.

Since benefits of PA include lower rates of all-cause mortality, and morbidity from conditions such as breast cancer, current guidelines recommend participating in moderate PA for 150 min/week (ACS, 2017). This study reveals that barriers, including post-treatment symptoms, social support, and neighborhood safety, prevent AA BCSs from participating in PA and meeting PAGs and that many of the currently available PA interventions are ineffective and unsustainable. PA-related health disparities among AA BCSs warrant the need for innovative and culturally relevant approaches to promote PA in this population. The involvement of a breast cancer support group in the development of PAID has the potential to enhance PA among AA BCSs.

SCT states that portions of an individual’s knowledge acquisition may be directly related to observing others within the context of social interactions and experiences. SISTAAH Talk exemplifies a support system for affecting the health of AA BCSs. Similar projects involving community organizations that have demonstrated appropriate design, implementation, and efficacy in promoting PA among underserved populations include the Southeast Senior Physical Activity Network (SESPAN) and the Active Aging Community Task Force (AACTF) project, (Cheadle et al. 2010). These programs incorporate means of motivating people who are inactive; creating effective, culturally relevant programs for the target population; and sustaining research-tested programs in community settings. Community engagement in developing a PA intervention will likely address physical inactivity and inequity among AA BCSs.

## Figures and Tables

**Table 1:** FGD-identified barriers and recommended strategies

Theme	Comments	Strategies

**Individual** Post-treatment symptoms	“With lymphedema and neuropathy, is it [exercise] safe?” “After radiation, I am concerned about how exercise will affect my chest”	Promote discussion of post-treatment symptoms Address symptoms related to lymphedema, arthralgia, and neuropathy and exercise safety through discussion and take-home fact sheets Encourage testimonials from BCSs on dealing with treatment effects (e.g., chemotherapy, surgery, and radiation) and tiredness/fatigue Include components focused on self-efficacy to engage in PA Focus on BCS-selected exercises Encourage a graded approach to PA engagement (based on stage of readiness) Engage BCSs in role playing to combat feelings related to body image and comfort in completing PAs
Tiredness/Fatigue Post-treatment body image	“I am always tired and my energy is too low [to exercise]” “I am not about to expose myself to a bunch of skinny women…not for me” “I am uncomfortable working out in public. Once I loose some weight, maybe...” “[After a double mastectomy], I am uncomfortable participating [in exercise classes]”	
Co-morbidity	“With high blood pressure, I am not sure that I should exercise too much” “Loosing weight will help my diabetes, but I am not motivated to workout” “I know its good for me, with all of my health problems, I just don’t want to do it”	Emphasize non-weight health benefits (physical and mental health) to motivate PA engagement. Highlight improvements in cardiorespiratory fitness, muscle strength, fatigue, depression, anxiety, and overall quality of life from regular PA Discuss the benefits of PA in controlling blood glucose, blood pressure, and serum lipids (and related chronic disease indicators)
PA perceptions	“I get enough exercise cleaning my house, running after my kids, and working on the job” “Black women don’t workout because we worry about messing up our hair” “I am afraid that it [exercise] could make the swelling in my arm worse”	Provide feedback (via accelerometers) to BCSs related to *actual* PA completed Incorporate information in sessions and distribute fact sheets to address PA misconceptions Address issues related to hair care in education and discussion sessions, and provide practical tools as an incentive for enrollment (e.g., head wraps to protect hair from sweat) Invite hair care experts to discussion sessions to promote natural hair styles

**Interpersonal** Social support	“If I had someone with me, I would be more likely to get out and do something” “We need a way to encourage each other when we [SISTAAH Talk] are not together”	Pair BCSs with supporters (co-survivors) Include “talking points” on how to talk with intimate partners, family members, and friends about PA safety, barriers, and benefits Outline methods and encourage BCS/co-survivors to communicate between sessions (e.g., phone calls/text messages, sharing resources, and engaging in PA
Intimate partner concerns	“Every time I loose weight, my husband gets scared…he thinks my breast cancer is back”	
Family support	“When I tell my family that I am trying to lose weight, they ask, ‘why bother?’	

**Organizational** PA preferences	“We need [exercise] programs that work for everyone—not a ‘one-size-fits-all’ approach” “If it says *sistaah*, make it [the intervention] welcoming”	Promote collectivism by including images of AA BCSs on all curriculum materials, including take-home fact sheets Provide child care during intervention sessions Incorporate PA in all SISTAAH Talk activities Focus on easy-to-achieve exercises Individualize approach to exercise sessions based on levels and stage of readiness for change Include PA preferred by AA BCSs
Monetary costs	“Whatever we choose, make sure it does not cost too much” “Most of us can’t afford to join a gym”	
Cultural Appropriateness	“Design the program with ‘us’ in mind” “Include things like gospel music or jazz” “Make sure it [the intervention] looks like its made for Black women”	

**Community** Lack of resources	“There are no resources in my neighborhood [gym, YMCA, free exercise classes]”	Provide guidance to SISTAAH Talk leadership in addressing inadequate community-level resources for PA Recommend completion of PA before or after work in safe, comfortable (e.g., temperature friendly) environments, providing examples Suggest engaging in PA with family and friends Provide a list of safe, free/low cost neighborhood-specific PA resources
Safety concerns	“With no street lights where I live, its not safe to go outside at night” “Even young children will point, stare, and harass me when I walk on the street”	
Weather	“Most of the year, it’s way too hot to exercise outside?	

**Table 2:** Theory-based content of PAID sessions

Number	Title	Content	Theoretical Component
1	What’s in it for me?	Program requirements; PA and cancer prevention guidelines	Outcome expectancy; self-efficacy; self-monitoring
2	Taking control	Introduction to walking—benefits and barriers	Social support; self-monitoring
3	Keeping score	Setting Specific, Measurable, Achievable, Realistic and Timely (SMART) goals and developing action plans	Goal setting; self-efficacy; self-worth; feedback
4	Know your body	PA and breast cancer recurrence	Social support, feedback, self-monitoring
5	Stay beautiful, stay alive	Addressing negative outcome expectancy related to fatigue, physical functioning, and hair	Outcome expectancy
6	Lifestyle and breast cancer risk	Dietary intake, PA, tobacco and alcohol use, stress management	Self-efficacy; social support
7	More *good* than harm	Challenges to PA; how to enjoy PA and improve attitude; PA safety	Methods for self-monitoring, behavioral cues, identifying and overcoming barriers; outcome expectancy
8	Keeping the faith	Maintaining walking behaviors	Self-monitoring; problem solving; stimulus control
9	Strong woman	Introduction to strength training—benefits and barriers	Self-efficacy
10	One day at a time	Balancing daily challenges with maintaining one’s health	Self-efficacy; social support
11	Tricks that stick	Strategies for increasing daily physical activity	Stimulus control; problem solving
12	Woman in the mirror	Review of preference for heavier ideal weight, incorrect assessment of normal weight, and satisfaction with body size	Self-evaluation and assessment of progress toward SMART goal
13	What’s love got to do with it?	Promoting self-care	Self-efficacy; self-esteem; social support
14	In my hood	Addressing safety and support; controlling the environment	Self-monitoring; problem solving; social support
15	Stay in the game	Review of PA and cancer prevention guidelines	Self-efficacy; self-monitoring; social support
16	Keeping the faith	Maintaining strength training behaviors	Self-efficacy; self-monitoring; social support
17	Mind over matter	Introduction to yoga–benefits and barriers	Outcome expectancy; problem solving
18	Slim down	Weight control	Self-monitoring; outcome expectancy; problem solving
19	Restoration	Sleep, meditation, rest; grocery shopping tour; guided discussion	Self-efficacy; self-monitoring; social support
20	On the run	Finding everyday opportunities to increase PA	Self-efficacy; self-monitoring; social support
21	It all works together	Review of cancer prevention and lifestyle—diet, PA, stress reduction	Goal setting; problem solving; outcome expectancy
22	Get moving to better health	PA benefits for BCSs	Self-monitoring; stimulus control
23	Keeping the faith	Maintaining yoga/Pilates behaviors	Self-efficacy; self-monitoring; social support
24	Looking back and moving forward	Celebration and strategies for maintenance	Outcome expectancy; self-efficacy; self-monitoring; social support
